# Modeling RNA polymerase competition: the effect of σ-subunit knockout and heat shock on gene transcription level

**DOI:** 10.1186/1745-6150-6-3

**Published:** 2011-01-21

**Authors:** Vassily A Lyubetsky, Oleg A Zverkov, Lev I Rubanov, Alexandr V Seliverstov

**Affiliations:** 1 Institute for Information Transmission Problems of the Russian Academy of Sciences (Kharkevich Institute), 19 Bolshoy Karetny per., Moscow, 127994, Russia

## Abstract

**Background:**

Modeling of a complex biological process can explain the results of experimental studies and help predict its characteristics. Among such processes is transcription in the presence of competing RNA polymerases. This process involves RNA polymerases collision followed by transcription termination.

**Results:**

A mathematical and computer simulation model is developed to describe the competition of RNA polymerases during genes transcription on complementary DNA strands. E.g., in the barley *Hordeum vulgare *the polymerase competition occurs in the locus containing plastome genes *psbA*, *rpl23*, *rpl2 *and four bacterial type promoters. In heat shock experiments on isolated chloroplasts, a twofold decrease of *psbA *transcripts and even larger increase of *rpl23*-*rpl2 *transcripts were observed, which is well reproduced in the model. The model predictions are in good agreement with virtually all relevant experimental data (knockout, heat shock, chromatogram data, etc.). The model allows to hypothesize a mechanism of cell response to knockout and heat shock, as well as a mechanism of gene expression regulation in presence of RNA polymerase competition. The model is implemented for multiprocessor platforms with MPI and supported on Linux and MS Windows. The source code written in C++ is available under the GNU General Public License from the laboratory website. A user-friendly GUI version is also provided at http://lab6.iitp.ru/en/rivals.

**Conclusions:**

The developed model is in good agreement with virtually all relevant experimental data. The model can be applied to estimate intensities of binding of the holoenzyme and phage type RNA polymerase to their promoters using data on gene transcription levels, as well as to predict characteristics of RNA polymerases and the transcription process that are difficult to measure directly, e.g., the intensity (frequency) of holoenzyme binding to the promoter in correlation to its nucleotide composition and the type of σ-subunit, the amount of transcription initiation aborts, etc. The model can be used to make functional predictions, e.g., heat shock response in isolated chloroplasts and changes of gene transcription levels under knockout of different σ-subunits or RNA polymerases or due to gene expression regulation.

**Reviewers:**

This article was reviewed by Dr. Anthony Almudevar, Dr. Aniko Szabo, Dr. Yuri Wolf (nominated by Dr. Peter Olofsson) and Prof. Marek Kimmel.

## Background

Plastids are semiautonomous organelles of plants also containing an independent transcription system. In plastids of plants and algae transcription is carried out by RNA polymerases of several types: one or two phage type polymerases (NEP) and one eubacterial type polymerase (PEP). NEPs are mono-subunit nuclear-encoded polymerases binding NEP-promoters, while PEPs are multi-subunit polymerases encoded in plastids and binding PEP-promoters. To initiate transcription, PEPs require one of σ-subunits encoded and regulated in nucleus. The intensity of RNA polymerase holoenzyme binding to a PEP-promoter and transcription initiation depend on the type of σ-subunit [1]. The "intensity" is the frequency of the polymerase binding to an accessible promoter, which is not preoccupied by another polymerase or a factor. This is an example of a regulation system based on interaction between the plastome and nucleome. Recently σ-subunit-coding DNA sequences were characterized in plants; e.g., *Arabidopsis thaliana *was found to possess six σ-subunits, Sig1-Sig6. Some σ-subunits are more universal, such as Sig1, others are more specific, e.g. the Sig5 subunit for the light-regulated promoter of *psbD *[2]. NEP-promoters of different types are generally better studied than PEP-promoters, especially those requiring minor σ-subunits. In many cases the location of NEP- and PEP-promoters were not experimentally known and were identified *de novo *in our analyses of multiple alignments of relevant leader regions as described in [3].

The RNA polymerase competition is mainly implemented through either polymerases collision followed by transcription termination or blocking of promoters by already bound polymerases. Binding of the polymerase to the promoter is possible only if at the moment the promoter is not occupied by another polymerase or a factor. If promoters are located so close that their binding is stoichiometrically mutually exclusive, polymerase competition also occurs. Of basic importance are the transcription initiation (especially applies to PEP) and interaction of the polymerase with nucleic acid secondary structures and protein factors. Simultaneous modeling of numerous PEP and NEP bindings and movements allows to interpret the published quantitative experimental evidence.

Transcription levels of many genes were compared between a *sig4*-knockout mutant and the wild type in *Arabidopsis thaliana *and other plants. Indeed, averaged ratios MT/WT of transcription level in mutant (MT) to that in the wild type (WT) and their dispersions in experimental assays were estimated for the cases of *sig4-*knockout [4] and *sig3-*knockout [5]. Analogous are heat shock and phytohormons level variation studies [6,7]. In heat shock experiments the estimates of the averaged HT/WT ratio and its dispersion (HT is the transcription level after heat shock, WT is that in the wild type) are formally similar with σ-subunit knockout studies. Response to heat shock is essentially different in native and isolated chloroplasts, as experimentally shown in [6]. The ratio of transcription level in shocked vs. intact isolated chloroplasts was assessed in [6] for several genes. In isolated chloroplasts gene transcription levels depend mainly on the polymerase elongation rate and the promoters' binding intensities, which reduces the effect of the nucleus to changes in σ-subunit concentrations.

**Figure 1:**
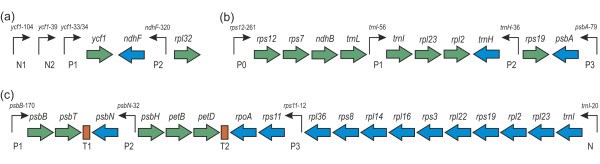
**Examples of loci**. **(a) **Locus 1 from *Arabidopsis*, **(b) **Locus 2 from *Hordeum*, **(c) **Locus 3 from *Arabidopsis*. PEP-promoters are designated with P, NEP-promoters - with N; T1 and T2 are predicted terminators. Positions of transcription initiation sites are given relative to start codons of the corresponding genes. DNA strand containing the promoter and the gene is marked with arrow and color, respectively.

Besides σ-subunit knockout and heat shock studies, modeling explains chromatogram data [8], however, with less accuracy. Chromatograms can be used to compare levels of the gene transcription from different promoters or before and after the knockout of phage type RNA polymerase. A lower accuracy is related to a poorer resolution of blotting methods, limited (a max. of two in [8]) assay replicates and ambiguous quantitative interpretation of chromatograms. For example, our analyses of chromatogram data in Figure 5c (see [8]) show that different promoters produce different transcription levels of gene *ycf1 *(ref. Figure 1c): RpoTp-dependent *ycf1*-39 is more effective compared to RpoTmp-dependent promoter *ycf1*-104, and is twice as effective as PEP-dependent *ycf1*-34/33. This data is in good agreement with the model prediction. Under RpoTp knockout (when promoter *ycf1*-39 is not functioning) the transcription level from *ycf1*-104 adheres to the same level, and from *ycf1*-34/33 it rather increases. We do not discuss the RpoTp knockout in this publication.

To tune model parameters we used evidence from independent studies. Namely, the effects of PEP-promoter mutations on the intensity of Sig1-3 subunits binding [9], phage type RpoTp-promoter mutations on NEP binding intensity [10], other studies of PEP- and NEP-dependent promoters in plastids [8,11,12] and Sig5-dependent promoter of gene *psbD *[13].

This study takes the first step toward modeling the RNA polymerase competition process. We predict intensities of the polymerase-promoter binding in good correspondence with experimentally measured changes of gene transcription levels, and propose a heat shock response mechanism in isolated chloroplasts. Polymerase binding intensities per se are not yet measured experimentally, but their predicted values do not contradict available indirect biological data (ref. to sub-section 5 under Methods).

A mathematical and computer model is developed to describe the competition of RNA polymerases during genes transcription on complementary DNA strands. The model predictions are in good agreement with the above described experimental data (knockout, heat shock, chromatogram data, etc.). The model is applied to estimate intensities of binding of the holoenzyme and phage type RNA polymerase to their promoters using data on gene transcription levels, as well as to predict characteristics of RNA polymerases and the transcription process that are difficult to measure directly, e.g., the amount of transcription initiation aborts, etc. The model is used to make functional predictions, e.g., heat shock response in isolated chloroplasts and changes of gene transcription levels under knockout of different σ-subunits or RNA polymerases or due to gene expression regulation.

## Methods

### 1. Introduction to modeling RNA polymerase competition

A locus is a region of double-stranded DNA with genes and promoters on both strands. Consider a locus, e.g., the one described in sub-section 2. For simplicity, assume that it does not receive elongating RNA polymerases from *outside*, i.e. all polymerases moving within the locus have initiated at one of its known promoters. After initiation the polymerase moves at a certain rate in one of the directions. Each promoter is characterized by number *λ*, the intensity of attempts to bind one of the surrounding polymerases. An attempt to bind the promoter is considered "successful" (i.e. the transcription is initiated) if the promoter is not occupied by another polymerase or a transcription factor. In other informal words, *λ *is a frequency ("conditional intensity") of binding attempts to the promoter when it is not occupied by another polymerase or a factor. It reflects the promoter sequence quality and corresponding polymerase σ-subunits availability. Formally, *λ *is the parameter of a Poisson stochastic process. Henceforth, the "intensity" stands for the frequency of binding attempts to a fixed promoter but under the assumption of no competition for it at the moment of attempt, and full availability of σ-subunits.

When two polymerases moving along complementary strands collide, they detach and transcriptions terminate at both strands. If a moving polymerase interacts with a cross-hairpin, the transcription also terminates with a certain probability *p *(the hairpin-specific parameter).

Some of the model parameters are fixed (estimated directly or indirectly from the experiment), others are varied in the modeling. Among the fixed are positions of genes and promoters on the DNA strands, among the varied are the above mentioned binding attempt intensities. "Model 1" is referred to when the expression levels of all genes within a locus are estimated with all model parameters known or fixed arbitrarily. Model 1 solves a nontrivial problem of building dynamically in time a profile of positions of up to thousands of polymerases transcribing a locus simultaneously, and also calculates a total number of completed transcriptions of each gene. "Model 2" is an extension of Model 1, in which some parameters are unknown and estimated in modeling by optimization, while the others are fixed. The terms "Model 1" and "Model 2" are conventional and refer to the two modes of one model.

The optimality criterion in Model 2 is maximal approximation of gene transcription levels known from the experiment (knockout, heat shock, chromatogram data, etc.); or, in other words, the simultaneous minimization of discrepancies between the transcription level vector obtained in the model and each of the vectors estimated in different experimental assays (arguments of all discrepancies are unknown parameters). Importantly, conditions of these assays can be reproduced in our modeling. In terms of computer science, Model 2 extends Model 1 by solving the "inverse problem". A nontrivial property of Model 2 is its ability to estimate unknown parameters, e.g., binding attempt intensities, such that transcription levels are predicted within experimental error for virtually all relevant experimental data. A mathematical description of Model 1 is given in sub-section 3, and its computer implementation - in sub-section 4. The ends of these sub-sections provide reasoning for the extension to Model 2 in terms of multicriteria minimization of the discrepancies as functions of binding attempt intensities. Further, in the Results section we compare gene expression levels predicted under promoter-specific binding attempt intensities estimated in Model 2 with known experimental data; intensity values are given and discussed.

### 2. Examples of loci

We exemplify our model on several loci.

#### Locus 1 in *Arabidopsis*

(Figure 1a): N1-N2-P1-*ycf*1-(*ndhF*-P2)-*rpl*32, where P1 = *ycf1*-33/34, P2 = *ndhF*-320, N1 = *ycf1*-104, N2 = *ycf1*-39. In parentheses are structures on the complementary strand. Henceforth, PEP-promoters are designated with P, NEP-promoters - with N. Note that the plastome contains two copies of the N1-N2-P1-*ycf*1 region, in one of which the short *ycf*1 gene is actually the beginning of the long *ycf*1 gene; the two copies are surrounded by quite different neighborhood. The *ycf*1 transcription level is a sum of two transcriptions.

For the first copy, N1 and P2 are preceded by highly transcribed genes on the complementary strand that virtually blocks polymerase access to this region. For the second copy, N1 is also preceded by highly transcribed genes on the complementary strand, and *ycf1 *is followed by a long operon on the same strand, which makes this copy of the region virtually independent of external promoters. This locus was studied in σ-subunit Sig4 knockout experiments under temperature +23°C.

#### Locus 2 in *Hordeum*

*Hordeum vulgare *possesses two regions. The first region (Figure 1b): P0-*rps12*-*rps7-ndhB-trnL*_CAA _-P1-*trnI*_CAU _-*rpl23*-*rpl2-*(*trnH*-P2)-*rps19*-(*psbA*-P3); and the second region: P0-*rps12*-*rps7*-*ndhB*-*trnL*_CAA _-P1-*trnI*_CAU _-*rpl23*-*rpl2*-(*trnH*-P2)-*rps19-...-rps16*, where P0 = *rps12*-261, P1 = *trnI*-56, P2 = *trnH*-36, P3 = *psbA*-79 are PEP-promoters. For the first region, polymerases initiate from P0 and P1, and from P2 and P3 - on the complementary strand; the second region lacks gene *psbA *and its promoter P3. For both copies, P0 is preceded by actively transcribed tRNA genes on the complementary strand that virtually blocks P0 from the upstream. In the first region, P3 is preceded by genes transcribed in the same direction, therefore a cumulative transcription from P3 is considered, i.e. with polymerases initiated at this promoter and the promoters upstream on the complementary strand. The second region adjoins the 5'-end of a large operon on the same strand that blocks the *trnH *transcription initiation from anywhere upstream of P2. This locus was studied in heat shock experiments: plants were grown for 6-7 days at 21°C and abruptly exposed at 40°C for 1.5 hr. Control plants were not exposed. During next 0.25 hr under 25°C the amount of complete transcripts was estimated with respect to the control plants. As the transcription level of *rpl23 *and *rpl2 *genes was measured cumulatively, the same was computed in the model.

#### Locus 3 in *Arabidopsis*

(Figure 1c): P1-*psbB*-*psbT*-T1-(*psbN*-P2)-*psbH*-*petB*-*petD *-T2- (*rpoA*- *rps*11-P3- *rpl*36- *rps8*- *rpl14*-*rpl16*-*rps3*- *rpl22*-*rps19*-*rpl2*-*rpl23*-*trnI*-N), where P1 = *psbB*-170, P2 = *psbN*-32, P3 = *rps11*-12 are PEP-promoters; N = *trnI*-20 is a NEP-promoter; T1 and T2 are terminators (likely cross-hairpins in DNA) predicted by the model in the regions T1 = *psbT *+ 22...*psbN*-1, T2 = *petD *+ 47...*rpoA*-139. The highly transcribed *slpP *gene is located upstream of P1 on the complementary strand, and active gene *ycf2 *is located downstream of N on the positive strand, in which situation the locus is virtually not transcribed from the outside. The locus was studied in Sig3 and Sig4 subunits knockout experiments at +23°C. The knockout was modeled for Sig3 and Sig4 under the same values of the polymerase-promoter binding intensities as in the wild type.

### 3. Model description

A locus can be transcribed by several polymerases at a time, which bind their promoters and move along the strands, possibly in the opposite directions. For each promoter the model uses the parameter of the RNA-polymerase-promoter binding attempts *intensity*. If not known experimentally, the parameter is estimated in the model. Time intervals between the attempts are described as a Poisson process, with an attempt being successful if at this moment the promoter is not occupied by another polymerase or a factor. Thus, each NEP- and PEP-promoter (the latter linked to a fixed group of σ-subunits) is assigned a Poisson process with parameter *λ*. The following groups were used: all σ-subunits and all σ-subunits but one knocked-out. In case of Locus 1 Sig4 is knocked-out, in case of Locus 3 Sig3 or Sig4 is knocked-out. Locus 2 was modeled under heat shock with all σ-subunits. Thus, each NEP-promoter is modeled as a stochastic process that defines the time interval between the attempts of NEP binding. The time is calculated as -(ln ξ)/*λ*_*N*_, where *ξ *is uniformly distributed random variable between 0 and 1. Parameter *λ*_*N *_is the desired value for the promoter. Analogously, stochastic processes are defined for each PEP-promoter. The time interval is again calculated as -(ln ξ)/*λ*, where *λ *is *λ*_*P *_if PEP is taken with all σ-subunits, and *λ *is *λ*_4 _if PEP is taken with all σ-subunits without the knocked-out Sig4. Sig4 is introduced for Locus 1, for Locus 3 Sig3 or Sig4 are instead introduced as in knockout experiments; *λ*_*P *_and *λ*_4 _(Locus 1) or *λ*_*P *_and either *λ*_3 _or *λ*_4 _(Locus 3) are pairs of parameters in modeling PEP-promoters. To be concise, here and forth all *λ*'s are called *binding intensities *of the corresponding promoters. Importantly, intensities estimated for the wild type are used further unchanged for modeling with different experiments in the same or close species. Intensities have dimension s^-1 ^(inverse seconds). If the 3'-sides of two polymerases occupy the same nucleotide, the model assumes that both elongations are terminated. If one polymerase (of the two transcribing the same strand one after another) moves faster, it is forbidden to outrun the slower polymerase. Modeling the elongation process requires parameters *ν*_*N *_and *ν*_*P *_of elongation rate of NEP and PEP, respectively. The rates depend on temperature, DNA nucleotide composition, and secondary structure of RNA that forms during transcription [14,15]. The results below were obtained under the assumption of a constant rate (at a fixed temperature) not affected by RNA structures, i.e. the elongation is currently modeled as a deterministic process.

Each transcription factor *F *is described by a similar stochastic process with parameter *λ*_*F*_, which defines the time interval between attempts of the factor to bind its DNA site. An attempt is successful if at this moment the site is not occupied by a polymerase, other factor, etc. Each terminator (a cross-hairpin in DNA) is associated with a Bernoulli random quantity (with probability parameter *p*) that describes transcription termination at a nucleotide of the hairpin's shoulder.

When a PEP-promoter is bound, first the initiation abort, then elongation are modeled. Modeling the abort process requires to set the amount of unsuccessful initiation attempts and the length of each abortive RNA, which are estimated in the model as follows. Time *t *of the abort process duration is defined as *t *= -(ln *ξ*) ⋅ *t*_0_, where *t*_0 _is average abort process time (e.g., 0.4 s, ref. to sub-section 5). Total amount of unsuccessful initiation attempts *k *is defined as a maximum number of summands in the left side of inequality

(1) $$-(\ln\xi_{1}+...+\ln\xi_{i}+...+\ln\xi_{k})\leq (t\cdot v_{P})/r_{0}$$

that keep it true. Here *r*_0 _is the average length of an abortive RNA (e.g. *r*_0 _= 4, ref. to sub-section 5). Each *i*-th unsuccessful attempt produces RNA with integer length closest to value -*r*_0 _⋅ (ln *ξ*_*i*_). Thus, value -(ln *ξ*_*i*_) is the random correction of average time *r*_0_/*ν*_*P *_of one unsuccessful attempt, where *ν*_*P *_is the rate of PEP.

To model transcription levels under heat shock (Locus 2) several parameters were introduced based on experimental data: during time *t*_1 _the plant is at temperature *T*_1_; during time *t*_2 _a certain amount of chloroplasts is exposed to temperature *T*_2_, and the same amount of chloroplasts - to same *T*_1_; during time *t*_3 _both populations of chloroplasts are exposed to temperature *T*_3_, then the ratio of complete transcripts is estimated for several plastome genes in the shocked vs. control plastids [6]. In the experiment time *t*_1 _was 6-7 days (after 3 hrs the model reaches stationary condition and further increase of *t*_1 _has no affect on the result), *T*_1 _= 21°C, *t*_2 _= 1.5 hr, *T*_2 _= 40°C, *t*_3 _= 15 min, *T*_3 _= 25°C.

Time intervals between any successive events in the model were estimated from distributions of stochastic and deterministic (polymerase movement) processes described above and then summed up. Thus, each event can be described with a *modeled real time *from the common start of all modeled processes; the modeled real time of course does not coincide with the computation time.

Effectiveness of the developed algorithms allowed for high model performance, which is about one-two orders of magnitude faster than corresponding biological processes. It is important to obtain statistically robust results. Biological experiments can last for over days on many thousands living cells, which provides for a statistically robust estimation of transcription levels. *In silico *reaching this degree of averaging can be problematic, therefore in our modeling the averaging was done over numerous trajectories (independent model runs) on a high-performance computing cluster. Each trajectory predicts the number of transcripts of each gene in the locus; after averaging and accounting for the modeled real time the estimates of transcription frequencies (levels) are produced for each gene.

Unknown parameters (promoter binding intensities, etc.) were estimated either directly based on particular experimental data (ref. to sub-section 5) or calculated in the model based on experimental data not related to measuring transcription levels. The first case is referred to as Model 1, the second - as Model 2 in the above sub-section 1. Model 2 exploits the standard computer science technique of inverse solution, i.e. a multicriteria optimization. E.g., define $\bar{y}=f(\bar{\lambda })$, where $\bar{y}=\left\{y_{i}\right\}$ is a vector of all unknown gene expression levels in the locus, and $\bar{\lambda }$ is a vector of unknown binding intensities (Model 1 allows to compute $\bar{y}$ for any given $\bar{\lambda }$); all the other parameters are fixed. Model 2 computes solution ${\bar{\lambda }}^{*}$ as a minimum of value

(2) $$\underset{i}{\max}\left\{\left|y_{i}-y_{0i}\right|/\left((y_{i}+y_{0i})/2\right)\right\}$$

where ${\bar{y}}_{0}=\left\{y_{0i}\right\}$ is a vector of all gene expression levels in the locus estimated experimentally. In most cases, however, expression (2) cannot be used, as value ${\bar{y}}_{0}$ is not inferred in the experiment. Instead, the ratio of each gene expression levels under *two different conditions *is measured. Then, Model 2 computes solution ${\bar{\lambda }}^{*}$ analogously as a global minimum of

(3) $$\underset{i}{\max}\left\{\left|z_{i}-z_{0i}\right|/\left((z_{i}+z_{0i})/2\right)\right\}$$

where ${\bar{z}}_{0}=\{z_{0i}\}=\{{y^{\prime }}_{0i}/{y^{\prime \prime }}_{0i}\}$ is experimentally measured vector of gene expression level ratios under these two conditions, and $\bar{z}=\{z_{i}\}=\{{y^{\prime }}_{i}/{y^{\prime \prime }}_{i}\;\}$ is a vector of corresponding ratios obtained in the model.

Above a single experiment (single optimization criterion) is considered, e.g. a σ-subunit knockout. Usually several experiments can be considered simultaneously, which requires a multicriteria optimization. Here, solution variants are ranked according to each criterion independently, and the solution with the minimal *total rank *is chosen. Weighting coefficients are used to allow for the comparative contribution of each criterion; otherwise, expression (3) can be simply summed over several experiments.

The two approaches used in Models 1 and 2 were comparatively analyzed and produced similar results (ref. to the end of sub-section 5).

### 4. Model implementation

The core of the model is implemented as an event-driven automaton. In the model each event is assigned to a point of modeled real time which is calculated with high accuracy, such that the probability of two events to coincide is virtually null. This allows us to model aforesaid stochastic and deterministic processes in chronological order, i.e. sequentially rather than in parallel. When the model processes an event sequentially, a next event is planned, connected to the current event and inserted properly in the common event queue. Processed events are removed from the queue.

An example of a typical event is an attempt of RNA polymerase to bind the promoter. The attempt is either successful or not depending on the promoter occupancy, but in both cases a time point for the next attempt is calculated (ref. to sub-section 3) and inserted in the event queue.

Apart from allowing for an unlimited queue of events, the model core is a finite state automaton controlled by a fixed set of rules, which can be easily specified in the program. Some of the rules are mentioned above, e.g., if two polymerases transcribing different DNA strands collide both elongations stop. The comprehensive description of the program object model, supported event types, the standard set of rules, as well as usage examples and recommendations, are provided in the user's manual (in Russian) available from the program web page [16].

The program is written in C++ and developed in two flavors. The user-friendly GUI version allows the user to monitor transcription of all genes along a trajectory of modeling and to easily tune the model parameters. This version of the program and a 32-bit MS Windows executable, which also runs on Linux with Wine package installed, are freely available at the laboratory website. Another version of the program is implemented as a command line utility for Windows or Linux. It is designed for large-scale modeling on a high-performance cluster with MPI version 1.1 or above installed. In this implementation multiple trajectories are modeled in parallel with the same set of parameters, which allows for the averaging of results to achieve higher reliability and estimation of confidence (ref. to sub-section 3). The source code of this program version is available for free at the website [16] under the GNU General Public License version 3 or above. The program performance crucially depends on the number of modeled objects (genes, promoters, etc.). For a mid-size locus (about 10,000 bp, 10 genes, promoters, and other elements) the typical processing time of a trajectory with 24 hr modeled duration will be between 3 and 15 minutes on a 3GHz Pentium-like CPU core, i.e., approximately hundredfold faster than the modeled real time. Notice that modeling along such a trajectory usually processes about 100 million events, hundreds of which can be queued in the same time.

Due to the program performance, it can also be applied for the estimation of unknown parameters (Model 2), such as binding attempt intensities, by minimizing expression (3): first a narrow region around its global minimum ${\bar{\lambda }}^{*}$ is selected (the region may itself be of interest) and then searched exhaustively for the global minimum itself. Narrowing this region is based on a simple statement that the measured genes are transcribed in both control and the experiment (knockout or heat shock). Therefore, movement of polymerases along two strands should be dynamically balanced at regions where the competition takes place. Otherwise, primary transcription of one strand inhibits transcription of the complementary strand. This is roughly equivalent to additional condition

(4) $$\underset{j}{\max}\left|\sum_{i\in I_{j}^{+}}\lambda_{i}-\sum_{i\in I_{j}^{-}}\lambda_{i}\right|\leq D$$

where maximum is taken over all regions between competing promoters; $I_{j}^{+}$ and $I_{j}^{-}$ are sets of the promoters located on positive and negative strands, respectively, to the left and right of the *j*-th region; *D *is a locus-specific threshold, which was chosen between 0.1 and 0.8 as a result of our computer experiments. Despite its simplicity, condition (4) can effectively reduce the exhaustion in Model 2 when the number of promoters with unknown binding intensity grows.

Computations for models 1 and 2 were conducted on supercomputer MBC-100K at the Joint Supercomputing Center of the RAS [17] using 2048 processors.

### 5. Model parameters

Sequence data was obtained from GenBank, NCBI. Multiple alignments were constructed with CLUSTAL 2.0.3 [18] in some cases to detect promoter sequences and validate crest-hairpins.

Multiple alignments of 5'-leader regions were constructed to predict unknown promoters when another angiosperm species was experimentally shown to have the promoter upstream orthologous genes [3]. The *psbA *promoter was thus predicted in barley (experimentally known in Arabidopsis, mustard and spinach) and the *psbB *promoter - in Arabidopsis (experimentally known in spinach). In cases when no data is available on promoters upstream orthologous genes in angiosperms, they were sought for by querying the 5'-leader regions against known chloroplast promoter consensuses as described in [3]. Analyses of multiple alignments validated the presence of cross-hairpins in DNA, which were predicted in our studies by modeling the polymerase competition.

PEP elongation rates at different temperatures correspond to those of the RNA polymerase in *E. coli*, as the subunits of these polymerases are close homologs [2]. Two linear correlations of the elongation rate vs. temperature [19,20] and a direct estimate of 42.5 nt/s at 37°C [21] are known for *E. coli*. Correlation with higher values from [20] was disputed in [19,21] as being systematically biased. Therefore, the correlation from [19] was adopted. It shows that at 21°C (normal growing temperature for barley) and 23°C (for Arabidopsis) the rates are 9.2 and 12.1 nt/s, respectively. During heat shock (Locus 2) the temperature was elevated to 40°C and then dropped to 25°C, which produced the rates of 36.8 and 15 nt/s. The replication fork rate in *E. coli *is 1500 nt/s, which we assumed to be the maximal NEP rate. The minimal NEP rate was set as the ribosome elongation rate of 45 nt/s. The authors are unaware of experimental data on the NEP rate in higher plants. The NEP rate has little impact on the results, because quickly after start the model predicts a dense chain-like movement of PEPs and NEPs. As a following-up polymerase cannot outpace another on the strand, most NEP rates are limited by PEP rates. Knowledge of NEP rate can improve the model accuracy. The model implementation enables one to vary NEP and PEP rates and other parameters widely.

The PEP size is set as that of the bacterial polymerase, NEP size - as that of the phage T7 polymerase. Studies of *E. coli*, *Thermus thermophilus *and chloroplasts of *Sinapis alba *with footprinting [22], kinetic [23], X-ray structure analyses [24] and promoter region mutagenesis [9] produce the following estimates: 35 nt (from -15 to +20 positions relative to the transcription initiation site) for the core-enzyme, 29 nt (from -44 to -16) for the DNA region shielded by the holoenzyme with a σ-subunit but not the core-enzyme; which gives an estimate of -44 to +20 for the holoenzyme. The -44 corresponds to the promoter size from a small region before the '-10' box of a PEP-promoter to a small region after the '-35' box of the same promoter. Considering the binding of α-subunits [25], the holoenzyme footprint can be extended upstream to position -60.

The promoter size experimentally identified in mutagenetic studies for the phage T7 NEP ortholog in chloroplasts of tobacco is -14 to +1 relative to the transcription initiation site [10]. The -15 position indicates a slight effect on the promoter quality [10]. Footprinting studies suggest that 15 nucleotide positions on the DNA strand are shielded by NEP; or, from another experiment, that 11 nucleotides are unpaired [26]. The estimate of 15 nucleotides was obtained in X-ray structure analyzes of the phage T7 polymerase [27]. The model assumes the NEP size to be -15 to +1 or better -15 to +4.

To estimate intensities of the PEP holoenzyme attempts binding to the promoter we used experimental data on the polymerase binding to the optimal promoter of gene *rrn *in *E. coli *with subsequent extrapolation of the estimate to the only and optimal promoter of gene *psbA *in Arabidopsis and then - to the PEP-promoters under study. In *E. coli *it is obtained based on experimental data on the amount of ribosomes, replication time and the number of rRNA gene copies: under good conditions of 37°C and 40 min generation time one cell contains 18700 copies of 23S rRNA, 16S rRNA and 5S rRNA each [28]. Simple calculations produce about 0.9 s between initiations at average. Therefore, the upper bound of intensity can be estimated as 1.113 s^-1^, which allowed us to set parameter *λ *within this upper limit. To infer binding intensity more accurately, the common time of 0.9 s is to be shared between the binding and the abort processes; e.g., 0.9 = 0.5 + 0.4. In modeling various proportions of times were estimated with the step of 0.1 s (e.g., the above mentioned abort time 0.4 s). In this example the binding intensity for optimal PEP-promoter *psbA*-77 of gene *psbA *in Arabidopsis and barley is 0.5. Now consider, e.g., promoters of genes *ycf1*-33/34 and *ndhF*-320 in Locus 1. The intensity of 0.5 in *psbA *is weighted with a reducing coefficient to reflect a lower quality of *ycf1*-33/34 and *ndhF*-320 promoters with respect to *psbA*-77, as follows from the experimental data on the effect of *psbA*-77 promoter mutations on the binding intensity in chloroplasts of mustard [9]. Note that in all photosynthesizing higher plants the *psbA *promoter is highly conserved [3,29]. The resulting intensities are: 0.09 s^-1 ^for *ycf1*-33/34, and 0.15 s^-1 ^for *ndhF*-320.

Estimation of the number of unsuccessful attempts requires, besides the average time *t*_0 _of the abort process, the knowledge of average length *r*_0 _of abortive RNA. A DNA∙RNA hybrid has length of about 9 bp, or even less because of σ-subunit binding [23,25]. Therefore, a single abortive RNA has length between 1 and 8-9 nt; various values were explored in modeling (in particular *r*_0 _= 4).

Importantly, parameter inference in the model 2 based on multicriteria optimization produces values similar to those estimated above from experimental data *not related *to transcription levels. An illustration is binding intensities of PEPs in Locus 1. Their above estimated values are 0.09 s^-1 ^for *ycf1-33/34*, and 0.15 s^-1 ^for *ndhF-320*, while the parameter optimization in the model 2 produces the values of 0.037 s^-1 ^and 0.093 s^-1^, respectively, which shows that the predictions are within the same order of magnitude.

## Results

The computer model was applied to study the RNA polymerase competition in a number of loci, for which a good agreement was obtained with experimental data on σ-subunit knockout, heat shock and other studies of plastids referred to in Introduction. The results are described for the three loci from sub-section 2 of Methods as being analogous with cases of other loci. Table 1 contains parameter values used to obtain results shown in Tables 2-3.

**Table 1:** Model parameters (except binding intensities) used to obtain results in Tables 2-3

Description	Parameter, condition
Core-enzyme position on DNA	[-15, +20] nt
Holoenzyme position on DNA	[-44, +20] nt
Phage-type RNA polymerase position on DNA	[-15, +4] nt
PEP elongation rate	9.2 nt/s at 21°C
	12.1 nt/s at 23°C
	15.0 nt/s at 25°C
	36.8 nt/s at 40°C
NEP elongation rate	45 nt/s at 23°C
Average duration of the abortive process	0.4 s
Average length of abortive RNA	4 nt
Modeled real time (Loci 1, 3)	24 hr
Duration of the heat shock (Locus 2)	1.5 hr
Aftershock time (Locus 2)	0.25 hr

Positions are given relative to the transcription initiation site.

**Table 2:** Comparison of gene transcription level ratios obtained in the model and experiment for Locus 1 (*Arabidopsis*) and Locus 2 (*Hordeum*)

Gene	Experiment	Model
Locus 1 (*Arabidopsis*)	*sig4 *knockout, MT/WT	
*ycf1*	0.73 ± 0.04	0.76 ± 0.01
*ndhF*	0.43 ± 0.10	0.47 ± 0.19
*rpl32*	1.52 ± 0.06	1.55 ± 0.02

Locus 2 (*Hordeum*)	Heat shock, HT/WT	
*rpl23*-*rpl2*	2.15/2.69	2.64 ± 0.02
*psbA*	0.53/0.55	0.54 ± 0.04

Standard deviations are provided where applicable. Values separated by a slash in the second column for Locus 2 are the results of two independent heat shock studies. The following binding intensities were used: N1 = 0.003, N2 = 0.054, P1 = 0.010/0.037, P2 = 0.050/0.093 (Locus 1) and P0 = 0.2, P1 = 0.9, P2 = 0.3, P3 = 0.1 (Locus 2), ref. to the Results. Other model parameters were fixed at the values shown in Table 1.

**Table 3:** Comparison of gene transcription level ratios MT/WT obtained in the model and *sig3 *and *sig4 *gene knockout experiments for Locus 3 in Arabidopsis

Gene	*sig3*-knockout	Model (*sig3*)	*sig4*-knockout	Model (*sig4*)
*psbB*	1.02 ± 0.36	1.27 ± 0.12	0.69 ± 0.19	0.84 ± 0.11
*psbT*	0.98 ± 0.25	1.30 ± 0.12	0.96 ± 0.15	0.85 ± 0.11
*psbN*	0.49 ± 0.46	0.41 ± 0.12	1.03 ± 0.02	1.02 ± 0.19
*psbH*	1.31 ± 0.05	1.28 ± 0.12	1.01 ± 0.08	0.83 ± 0.11
*petB*	0.91 ± 0.15	1.09 ± 0.11	0.87 ± 0.29	0.83 ± 0.11
*petD*	0.92 ± 0.09	0.89 ± 0.10	0.81 ± 0.21	0.81 ± 0.11
*rpoA*	0.94 ± 0.14	0.82 ± 0.20	0.79 ± 0.11	1.01 ± 0.14
*rps11*	0.92 ± 0.33	0.90 ± 0.21	0.98 ± 0.31	1.01 ± 0.13
*rpl36*	0.88 ± 0.11	1.03 ± 0.21	1.54 ± 0.62	1.08 ± 0.18
*rps8*	1.11 ± 0.04	1.03 ± 0.21	0.83 ± 0.15	1.08 ± 0.18
*rpl14*	1.04 ± 0.15	1.03 ± 0.21	1.11 ± 0.02	1.08 ± 0.18
*rpl16*	1.09 ± 0.03	1.03 ± 0.21	1.18 ± 0.03	1.08 ± 0.18
*rps3*	1.24 ± 0.26	1.03 ± 0.21	1.25 ± 0.02	1.08 ± 0.18
*rpl22*	1.09 ± 0.13	1.03 ± 0.21	1.20 ± 0.12	1.08 ± 0.18
*rps19*	1.15 ± 0.50	1.03 ± 0.21	0.96 ± 0.07	1.08 ± 0.17
*rpl2*	0.94 ± 0.15	1.03 ± 0.21	0.95 ± 0.06	1.08 ± 0.17
*rpl23*	1.05 ± 0.04	1.06 ± 0.20	1.35 ± 0.33	1.10 ± 0.17

Termination probabilities are T1 = 0.25 and T2 = 0.25; binding intensities are P1 = 0.555/0.867/1.355, P2 = 0.075/0.227/0.284, P3 = 0.116/0.146/0.182 (*sig3*, *sig4 *mutants and the wild type, correspondingly), N = 0.116, ref. to the Results. Other designations as in Table 2.

In the study [4] the ratio MT/WT of gene transcription level in a *sig4*-knocked-out mutant with respect to the wild type was estimated for three genes in Locus 1. These results, as well as transcription level ratios with different promoters are well reproduced in our model under the following values of the holoenzyme or phage type polymerase binding intensities: N1 = 0.003, N2 = 0.054, P1 = 0.010/0.037, P2 = 0.050/0.093. Here and forth, *designations *of the binding intensity and the corresponding promoter are the same, and two numbers are given for PEP-promoters, with the *first number *being the binding intensity under knockout and the *second number *being the intensity in the wild type. Table 2 shows the experimental and predicted gene transcription level ratios under Sig4 knockout. The gene *ycf1 *transcription level ratio from promoters N2 and P1 is 1.7. This ratio was not estimated for promoters N1 and N2 in the model, however, the N1 binding intensity is 20 times lower than in N2, which suggests a similar difference in their transcription levels. Under RpoTp knockout (when N2 = 0) and P1 = 0.12, the model predicts a 2.5-fold increase in the *ycf1*-33/34 PEP promoter efficiency comparing to the wild type, which is in agreement with chromatogram data [8]. Theoretically it can be justified by the absence of non-linear interaction based on one- and three-dimensional diffusions between RNA polymerases that initiate transcription from NEP-promoter N2 and PEP-promoter P1 (Figure 1a). The model predictions and experimental data differ within experimental error (Table 2), which suggests a good agreement with biological reality.

In the study [6] the transcription level ratio was measured in chloroplasts before and after heat shock (21°C normal temperature and 40°C shock temperature) for some genes in Locus 2. Good concordance between the model and the experiment was obtained under the values of the holoenzyme binding intensities P0 = 0.2, P1 = 0.9, P2 = 0.3, P3 = 0.1. Table 2 shows the predicted and measured transcription level changes after heat shock for this locus. Differences are evidently within the experimental error. The expression level of gene *rps16 *(the second region) increases after heat shock both in the model and experiment. The increase is slightly higher than that predicted in [6].

**Figure 2:**
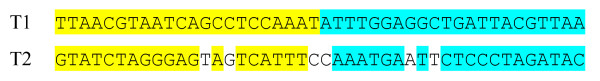
**Two putative terminators T1 and T2 in Locus 3 in *Arabidopsis***. Complementary bases are shown in color. The length of terminators is 44 bases, however sequences are different. Terminator T2 is an imperfect palindrome with three non-complementary pairs.

For Locus 3, the model predictions did not agree with the experimental data regardless of the choice of binding intensities, unless an additional assumption on the locus structure was made, which received, however, further support (ref. to Discussion). Namely, Model 2 was used to test different hypotheses of transcription termination by various factors hypothetically present in the locus, including cross-hairpins. The model results fell in *best *agreement with the experimentally measured gene transcription levels under the presence of two hypothetical terminators. Thus, Model 2 predicted two terminators as transcription termination factors, which were validated in analyses of multiple alignment of the corresponding DNA regions. The terminators are designated T1 and T2 in Figure 1c and are palindrome structures of 44 nt length (Figure 2). The conservativity of palindrome T1 and its role is described in [30] for a limited number of other species. Each terminator T1 or T2 is characterized by probability (*designated *T1 or T2) of elongation termination estimated in the model along with the promoter binding intensities. The probabilities are T1 = 0.25 and T2 = 0.25. The following intensities were predicted: P1 = 0.555/0.867/1.355 (for *sig3*, *sig4 *and the wild type), P2 = 0.075/0.227/0.284, P3 = 0.116/0.146/0.182, N = 0.116. The estimated transcription level ratios conform well with *sig3 *knockout experiments and the independent study of *sig4 *knockout in *Arabidopsis thaliana *[4], where transcription level ratios were measured before and after the knockout, as for Locus 1. Table 3 shows changes in gene transcription levels with and without Sig3 or Sig4 subunit knockouts obtained in the model and experiment; the values are similar. Particularly, the gene *psbB *transcription level is about 417 transcripts per hour (higher than in other genes), which well agreed with the experimental knowledge that it encodes a core apoprotein of photosystem II and, thus, must be highly transcribed.

## Discussion

Importantly, the model predictions for all loci conform with the results of knockout and heat shock experiments within the error range reported in the corresponding studies (Tables 2-3), as well as with chromatogram data from [8]. This attests that RNA polymerase binding intensities predicted in the model correspond well to measured gene transcription level changes. Particularly, PEP-promoter binding intensities estimated in experiments not related to knockout or heat shock are close to the model predictions (ref. to sub-section 5 under Methods). The values of other parameters used in the model neither contradict with known, however mostly indirect, biological evidence.

**Figure 3:**
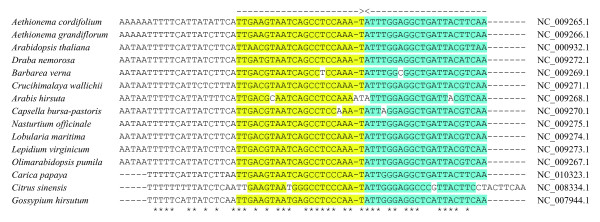
**Multiple alignment of putative transcription terminator T1 of genes *psbT *and *psbN *in chloroplasts of eurosids II**. Complete intergenic region is shown (in *Arabidopsis *its position is 74184-74248). Arrows and colors mark complementary shoulders in the palindrome. Asterisks mark conserved positions, dashes denote gaps.

For Locus 1 and gene *ycf1 *transcription level ratios were experimentally identified for its three promoters [8] and they are well reproduced in the model. For Locus 2 the predictions are in good agreement with the experimental data on transcription level changes during heat shock of isolated chloroplasts not affected by nuclear encoded heat shock proteins. For Locus 3 terminator T1 was predicted to exist between genes *psbT *and *psbN *adjoining the 3'-end of *psbN *in chloroplasts of *Arabidopsis thaliana*. It was validated on the multiple alignment of corresponding region to occur widely in plastids of plants and algae, see also [30]. In chloroplasts of eurosids II it is a palindrome structure of 44 nt length with the consensus TTGAMGTAATCAGCCTCCMAATATTKGGAGGCTGATTACKTCAA (Figure 3).

This palindrome is likely to fold into a cross-hairpin in DNA and function as a transcription terminator. An analogous transcription termination mechanism was earlier predicted in trailer regions of actively transcribed genes in Actinobacteria [31]. The palindrome might as well function as a cooperative factor binding site. Terminator T1 must be weak, as it precedes the highly transcribed polycistronic region that includes genes *petB *and *petD*, and indeed its predicted termination probability is low in our model.

Terminator T2 in the same locus has similar characteristics: it is located between genes *petD *and *rpoA *in chloroplasts of *Arabidopsis thaliana *and contains a palindrome of 44 bases length with coordinates 77719...77762 (whereas the complete intergenic region is 77673...77900 according to the GenBank annotation of NC_000932.1) and three non-complementary pairs (an imperfect palindrome). A multiple alignment of the T2 region is of the same quality as the T1 alignment. The two terminators can affect transcription in both directions. Despite being similar in length, putative terminators T1 and T2 are different in sequences, which suggests that corresponding palindromes are likely to form terminator hairpins due to base pairing.

The model allows to hypothesize a mechanism of cell response to knockout and heat shock (described above in detail), as well as some mechanisms of gene expression regulation based on RNA-polymerase competition.

## Conclusions

The mathematical and computer simulation model is developed to describe the competition of RNA polymerases during gene transcription on complementary DNA strands. The model is implemented for multiprocessor platforms with MPI and supported on Linux and MS Windows.

The model predictions are in good agreement with virtually all relevant experimental data (knockout, heat shock, chromatogram data, etc.). PEP-promoter binding intensities measured in experiments not related to knockout or heat shock are close to the model predictions. The model was used to make functional predictions, e.g., heat shock response in isolated chloroplasts and changes of gene transcription levels under knockout of different σ-subunits or RNA polymerases or due to gene expression regulation based on RNA polymerase competition. The model allows to hypothesize some mechanisms of cell response and gene expression regulation. The model is able to predict potential elongation termination factors and their localization.

It allows for tentative estimation of the properties of RNA polymerases and transcription process that are difficult to measure directly: the amount of transcription initiation aborts, the intensity of holoenzyme binding to the promoter in correlation with its sequence and the type of σ-subunit, etc.

## Competing interests

The authors declare that they have no competing interests.

## Authors' contributions

VAL and AVS proposed the model, estimated its parameters, chose source data and participated in the analysis of results. LIR participated in the model development and implementation: wrote software, performed the computations and participated in the data preparation and analysis. OAZ performed the statistical analysis and participated in the data preparation and the computations. AVS performed the sequence alignment and the search of promoters. VAL, AVS and LIR wrote the manuscript. All authors read and approved the final manuscript.

## Reviewers' comments

### Reviewer's report 1

Dr. Anthony Almudevar

Department of Biostatistics and Computational Biology, University of Rochester

The authors develop a model for transcription in the presence of competing RNA polymerases (involving polymerase collision followed by transcript termination). Competition is caused by occupation of a target (or nearby) promoter. A competition effect is reported as being observed in barley *(Hordeum vulgare) *based on various published perturbation experiments, including knockout experiments and heat shock experiments, which force two-fold alteration in gene expression levels.

In the model, transcription rate is mechanistically predicted from separate binding rates governing distinct Poisson processes, as well as other model components, such as transcription termination probabilities due to cross-hairpins. These parameters may be directly altered by experimental perturbation.

The authors define two models "Model 1" in which parameters are fixed, and "Model 2", which is an extension of Model 1 in which some parameters are unknown and estimated by model fitting (the inverse problem).

The model is applied to 3 cases (termed Locus 1,2,3) from *Hordeum *and *Arabidopsis*, for which either heat shock or σ-subunit knockout experimental data is available. Agreement of model predictions and experimental data is reported. Software is made available from the laboratory website.

1. The biological background and motivation seem good to me. It should be made clear, however, whether the cited experimental changes in gene expression are necessarily due to RNA polymerase competition, or whether there are other fundamentally different models which could conceivably explain these observations.

#### Authors' response

All discussed loci contain positionally close promoters on complementary strands, which initiate mutually interfering transcription processes (in the wild type, under knockout or heat shock). Therefore, the polymerase competition at these loci is objectively present (some are not included in the manuscript to meet the size limitations). Competition for the promoter is also detected, at least under some model parameterization and is expected from biological reality.

The model describes the competition in good agreement with versatile experimental evidence (knockout, heat shock and chromatograms). The authors are unaware of other models that explain these experiments. The biological explanation of heat shock response and gene expression regulation through RNA polymerase competition is also novel, as far as the authors are informed.

Apart from this, my main concerns are with the modeling.

### Concerns

2. An important issue with this kind of model is identifiability. Can we expect increasingly accurate resolution to a correct model given increasingly accurate data? Alternatively, is the model too complex for adequate resolution, so that any prediction success can be attributed to overfitting? These issues are not addressed in the paper, and so it is difficult to interpret the results.

#### Authors' response

In Model 2 the *solution*, i.e. a vector of promoter binding intensities and some other parameters, is found by best approximation of experimental *data*, largely on gene transcription level ratios. Importantly, the solutions are very few (always fewer than five-ten) in the space of many millions. Tests for robustness are not described in the manuscript but showed high model reproducibility and its robustness against the effect of minor variations of the predicted promoter binding intensities on gene transcription levels. "Global" parameters, e.g., the predicted NEP rate, were uniform across various loci.

Our model is largely non-linear, and direct collation of the number of "equations" (data) and "unknowns" (varied parameters in Model 2) is not informative. However, for locus 1 the experimental data and varied parameters were as follows: six different promoter binding intensities versus three gene transcription level ratios (under sigma-subunit knockout) plus experimental data on transcription level ratios from different promoters and data on NEP-promoter knockouts. With locus 3, twelve binding intensities and two transcription termination probabilities versus transcription level ratios of eighteen genes (under sigma-subunit knockouts). With locus 2, Model 2 was applied to approximate data on varying temperature in heat shock experiments. All predictions are supported by independent data, e.g., the presence of terminators is validated by multiple alignment, etc.

Finally, the model in fact describes the logic of a "multiparticle" system behavior, and the trial was to apply it to interpret all available data. In this respect, our results might be considered successful.

3. At the end of METHODS subsection 3, the authors state: "The two approaches used in Model 1 and 2 were comparatively analyzed and produced similar results (ref. to the end of sub-section 5)" The discussion referred to by the authors I found far too cursory and unconvincing, especially given the importance of the issue.

#### Authors' response

It seems important that parameter values obtained in Model 2 by approximating the experimental data on gene transcription level ratios (knockout, heat shock, etc.) also fall in agreement with independent experiments (described in sub-section 5). See also the second paragraph of the answer to concern 4.

Note that in the model the promoter binding intensity is set to be the same in the wild type and vary under knockouts, which significantly limits the solution space.

4. In the RESULTS section for the Locus 1 and 2 case, agreement between predicted gene expression levels, and those published in the literature is reported (Table 2) for a set of parametric intensities. It needs to be made clear the origins of these values (whether obtained from the sources described earlier, or estimated from Model 2 analysis), to assess if overfitting may be an issue. For the Locus 3 example, an additional model component (terminators) had to be introduced to yield accurate predictions. This new component was validated in further analysis. The authors refer here explicitly to Model 2. Again, we need to know if overfitting is an issue.

#### Authors' response

For all loci binding intensities were estimated from Model 2, see answer to concern 2.

The intensities described in the end of sub-section 5 and applied in Model 1 produce a poorer (however of the same order) fit to the experiment; these are crude estimates, and we chose to use the intensities and transcription levels obtained in Model 2, the corresponding ratios are shown in Tables 2-3.

All loci were tested for the presence of terminators and DNA-factor binding sites. Those were not found for loci 1 and 2; binding sites (if different from the terminator regions) were not found for locus 3.

**Figure 4:**
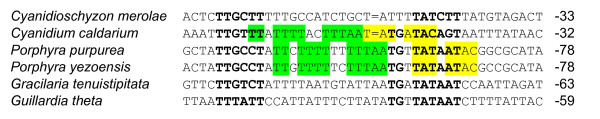
**Putative promoters upstream of gene *rps20***. In bold are the (-35) and (-10) boxes of the promoter and the (-10) box 5'-extension. Colored are conserved putative repressor binding sites. In the last column are distances to the start codon.

The model allows to hypothesize a mechanism of cell response to knockout and heat shock (described above in detail), as well as a mechanism of gene expression regulation based on RNA polymerase competition that in our opinion corroborates the model. Let us provide an example of gene regulation. In chloroplasts of Rhodophta we found the *glnB *5'-leader regions to contain conserved motifs with the consensus GTATyATA or TTAAAnnAAAAnAA (complementary regions are shown in Figure 4). These motifs contain the GTA triplet, the conserved core of the NtcA binding site in cyanobacteria. These regions can be hypothesized to bind NtcA in chloroplasts. Unlike the bacterial consensus, the putative NtcA binding site in plastomes is not palindromic: in cyanobacteria the GTA triplet is duplicated in the sense (the first palindrome shoulder) and antisense (the second shoulder) DNA strands thereby allowing for a better binding of the NtcA dimer. Gene *ycf28 *is orthologous to bacterial transcription factor NtcA from the Crp family that is characterized by the conserved PF00325 domain (Table 4, data from Pfam database). Although all rhodophyte plastomes possess *ycf28 *homologs (as per year 2010) the PF00325 domain is preserved only in those containing gene *glnB*, which is syntenically tied with *rps20*: *Cyanidium caldarium *(contains an *rps20 *pseudogene), *Porphyra purpurea *and *P. yezoensis*. We propose a hypothesis to explain this co-existence. Putative NtcA binding sites in the *glnB *5'-leader region overlap the divergently sited *rps20 *promoter (shown on the *rps20 *strand in Figure 4). The promoter conservativity cannot explain that of the regions, as their position varies relative to the promoter boxes. Importantly, the strand in between the regions and the *glnB *start codon contains neither the bacterial *σ*^70^-promoter nor other detectable promoter types. The *glnB *promoter boxes are not detected, but their lack might be compensated by transcription activator NtcA. This factor might also regulate the locus as a transcription repressor of the antisense promoter by relaxing the RNA polymerase competition. Note supportive evidence: in *Synechococcus *sp. PCC 7002, NtcA activates the transcription of gene *glnB*, which is syntenically distanced from *rps20 *thus precluding the polymerase competition. However, in rhodophytes it represses *rps20 *and activates the *glnB *gene. Note that both genes lack in Streptophyta. This subject is discussed in detail in [32].

**Table 4:** Crp family-specific PF00325 domains located close to C-termini of Ycf28 proteins in rhodophyte chloroplasts

Sequence	*E*	Begin	Domain	End
The domains consensus			LpmsLRqeIAdylGlTrETVsRlLtrLrekgLI	
*Cyanidioschyzon merolae*	0.9	114	WRLS-QASLARILGTSRAAIGQVLGDWKKQAWL	145
*Cyanidium caldarium*	0.00019	157	IYIS-QHDIASILSTTRSTITRLINQLRKDNII	188
*Porphyra purpurea*	0.00052	184	LTIT-HKVLAQIIGSNRVSITRIISKLIHTKFI	215
*Porphyra yezoensis*	0.0021	184	FTIT-HKILAQIIGSNRVSVTRILANLLKTKLI	215
*Gracilaria tenuistipitata*	> 1			

In rows, the Crp consensus is followed by the list of rhodophyte species with available chloroplast genomes. Individual domains are aligned against their consensus computed with Crp protein data from the Pfam database. The domain positions are relative to the protein N-terminus. E-values are shown for pair-wise alignments, the region with e-value > 1 is omitted.

5. There are several ways to approach this problem. Identifiability might be determined (or disproved) mathematically. Alternatively, prediction ability (of gene expression and unknown parameters) can be assessed using some empirical method (cross-validation or bootstrap), assuming that data replicates are available. Ideally, a model developed from one set of data can be applied directly to a new set, where homogeneity of parameters can be reasonably assumed.

#### Authors' response

The locus data are far too heterogeneous to be used for both inference and testing. Consider the three loci described in the manuscript. Although loci 2 and 3 contain several orthologs, their promoters differ due to chromosome rearrangements in different genomes. E.g., genes *trnI*-*rpl23*-*rpl2 *in locus 2 are transcribed from one of the PEP-promoters, while their orthologs in locus 3 - from the NEP-promoter. From the largely conserved *psbA *and *psbB *promoters, the former is absent in locus 3, the latter - in locus 2. Loci 1 and 3 in *Arabidopsis *differ in gene content.

It would be preferable to estimate promoter binding intensities based not on transcription level ratios but directly on the promoter nucleotide composition (e.g., its deviation from a taxon-specific consensus), concentration of preferred sigma-subunits, "topology" of the promoter neighborhood (DNA curvature, etc.), and Fourier coefficients of the DNA strand potential. The described model may be useful to achieve this.

6. In a related issue, are estimation errors available for the prediction values in Tables 2, 3? If this is not possible, the reasons should be discussed. In addition, could a summary of agreement be provided, especially for Table 3?

#### Authors' response

In response to this concern, Tables 2 and 3 now contain standard deviations of transcription level ratios obtained in Model 1 using binding intensities given in the legends and estimated in Model 2.

Loci 1 and 2 are found to behave very similarly in the model and experiment (changes in transcription levels are similar, refer to Table 2). In locus 3, the key genes (*psbN *and *psbH *for *sig3*-knockout and *psbB *for *sig4*-knockout) express similar transcription level changes in the model and experiment; other genes largely maintain the same levels before and after the knockout in qualitative agreement between the model and experiment.

The chi-square value exceeds 0.65 for some genes (e.g., *ndhF *in locus 1 and *psbA *in locus 2) but is lower for others. Statistical validation of the model, particularly the applicability of the chi-square criterion, is subject of a separate study and is likely to require more experimental replicates. Other statistical issues also remain open, e.g., some genes exhibit normal distributions of transcription levels before and after perturbations. Therefore, the level ratios estimated in the model are distributed similar to the Cauchy distribution, which has no expectation and infinite dispersion.

### Reviewer's report 2

Dr. Aniko Szabo

Division of Biostatistics, Medical College of Wisconsin

This is a fascinating paper that combines detailed understanding of biological processes with modern computational solutions. It describes a computational model of gene transcription that is based on modeling the actions of RNA polymerases including transcription initiation, elongation, and termination due to collisions. The model is implemented as a simulation software that can be used to predict gene transcription levels based on a set of parameters, as well as estimate some parameters based on information about gene transcription levels or their changes under knock-out or heat-shock conditions. The model relies on detailed understanding of the transcription process, and the region of interest, though an interesting example of the reverse process is also included: lack of fit to observed data led to new information about the structure of the gene.

While the paper does give a reasonable description of the mathematical model, the manual of the program available through the author's website is much more detailed and helpful (and longer). It would be worthwhile to highlight this point in the paper, especially if key parts of the manual could be translated into English.

#### 0. Authors' response

Manual in [16] contains a simplified description of the abort process. A full manual in English will supplement the model software publication.

Suggested changes:

1. Explain the choice of a Poisson process to model binding events. I agree that this is a good choice, but some justification in the paper would be useful.

#### Authors' response

The Poisson flow is used because a superposition of many independent stationary arbitrary flows is asymptotically approximated by a Poisson flow (the Poisson theorem).

2. In multiple places distributions of random variables are not given by their names, but rather based on their relationship to the uniform (0,1) distribution that is used to generate random samples. It would be much more helpful if, say, the distribution between consecutive events in a Poisson process were called exponential with rate/mean lambda instead of -ln(xi)/lambda. Similarly, the exponential distribution for the time of the abort process, the negative binomial (?) distribution for the number of unsuccessful initiation attempts, the geometric length of abortive RNA pieces, etc, should be named as such.

#### Authors' response

We assume that the distribution density of time lapses between polymerase binding attempts to the promoter is exponential distribution *λ *⋅ exp (-*λt*) with parameter *λ*. The times are modeled with -(ln *ξ*)/*λ *as described in the text. We assume that number *k *of abortive attempts is geometrically distributed with parameter *p*, where *p *is estimated from $\frac{1}{p}-1=\frac{t_{0}\cdot r_{0}}{\nu_{P}}$, which is modeled by inequality (1) as described in the text. The parameters are given in Table 1.

3. The description of the abortive initiation process is unclear, and appears to be different from the description in the manual (which is much clearer).

#### Authors' response

Parameter *t *is estimated from *t *= -(ln *ξ*) ⋅ *t*_0_, where *t*_0 _is one of the parameters given in Table 1; then *k *is found from inequality (1), other parameters in (1) are given in Table 1. See Authors' response 0 above.

4. The optimization criterions do not account for the fact that data-based estimates for the components of the y (or z) vector are known with differing precision. Perhaps a weighting scheme based on the inverse variances could be used. Additionally, for ratios relative difference might not be a good measure due to the lack of symmetry around 1. Suppose the true ratio is 2-fold, and the model predicts a 3-fold ratio. Then the absolute relative difference is |3-2|/2 = 0.5. Now for another gene the situation might be the inverse: the true ratio is 1/2 while the model predicts 1/3. The absolute relative difference for the second gene is |1/3-1/2|/(1/2) = 0.33, implying a better fit, while the two genes are arguably fitted equally well. I would suggest using log-ratios in criterion (3).

#### Authors' response

Introducing a logarithm may cause cases of taking a logarithm of a very small (or log0) or a very large value. Avoiding this again leads to asymmetry. Different functions tried in (3) produced largely similar solutions. In response to this remark, equation (3) is changed to contain (*z*_*i *_+ *z*_0__*i*_)/2 instead of *z*_0__*i *_in denominator, and results are re-calculated.

5. In the section describing the model parameters and the results sections it is somewhat hard to follow which values are fixed based on external considerations, and which are estimated to fit the observed data. It would be helpful if Tables 2 and 3 were accompanied with corresponding tables of fixed vs varying parameters (with their values).

#### Authors' response

The "global" parameter values are now provided in Table 1. Promoter binding intensities were varied to obtain results in Tables 2,3; while binding intensities, the terminator hairpin localization and size, and termination probability were varied to obtain results in Table 3, as now described accordingly in the legends.

6. The fact that for Locus 3 the original model did not fit and the inclusion of terminators was found to be necessary is very interesting. However some indication of the extent of the lack of fit and its improvement would be helpful.

#### Authors' response

In (3), the discrepancies between the prediction and experiment under presence or absence of additional termination factors are 0.35 and 0.72, respectively. When only the three key genes are considered (ref to response #4 AA), those are 0.20 and 0.72.

### Reviewer's report 3

Dr. Yuri Wolf

National Center for Biotechnology Information

(nominated by Dr. Peter Olofsson, Mathematics Department, Trinity University)

The manuscript by Lyubetsky et al. describes a detailed simulation model for chloroplast transcription system and reports very interesting results. The model has both explanatory (i.e. allows to formulate hypotheses on why particular transcription levels are observed in experiments) and predictive power (i.e. allows to predict the transcription levels under conditions of future experiments). As such, the results themselves and especially the software accompanying the paper are of considerable interest to the community.

Unfortunately the text suffers from the lack of systematic presentation of the basic concepts and assumptions of the model. The reader is left on her own to figure out what the authors mean upon the introduction of the term; explanation might (or might not) follow further down the text. Specific problems that caught my attention are listed below.

p. 3 and elsewhere. "intensity of holoenzyme binding". The authors introduce "binding intensity" in the paragraph and use it throughout the introduction without proper explanation. Only later it becomes apparent that it refers to frequency of binding attempts under assumption of no competition.

#### Authors' response

This term is now clarified properly in the text. Indeed, the "intensity" refers to the frequency of binding attempts to a fixed promoter, but under the assumption that at the moment of attempt it is not occupied any another polymerase or a factor, i.e. under no competition for the promoter at this moment, and full availability of σ-subunits.

p. 5. "Besides sigma-subunit knockout and heat shock studies, modeling explains chromatogram data [8], however, with less accuracy" - the relevance of "chromatogram data" is not clearly explained. If the authors are talking about measured transcription levels, the experimental technique used to obtain them probably matters little for the readers of this paper.

#### Authors' response

Chromatogram data from [8] require processing on bands measurements, as these are not originally provided. Lower precision is explained in the text.

p. 5. "this data is in good agreement with the model prediction" - there is no "model prediction" (or the model itself) yet to speak of. Comparison of the model prediction with the data belong to the Results or Discussion section.

#### Authors' response

The Introduction contains several phrases that describe the usage of experimental data further in the text.

p. 6. "Consider a locus, e.g. like one described in sub-section 2" - why are we forward-referenced to another sub-section here? It seems that a "locus" is one of the key concepts of the model - why it isn't defined explicitly instead of by example?

#### Authors' response

Corrected. A locus can be named any region of double-stranded DNA with genes and promoters on both strands. We considered loci with concurrent transcription on both strands to study polymerase *flows*. Cases with transcription initiation outside the locus are more difficult to interpret.

p. 6. "Each promoter is characterized by number lambda, the intensity of attempts to bind one of the surrounding polymerases" - again, lambda is one of the key parameters of the model. For some reason it is introduced three (and a half) times in one paragraph, each time slightly differently: "the intensity of attempts to bind...", "a frequency ('conditional intensity') of binding attempts" and "the parameter of a Poisson stochastic process". Neither of these become fully clear until an operational definition emerges in subsection 3.

#### Authors' response

«The intensity of attempts to bind» and "frequency ('conditional intensity')» were used to intuitively explain that only some of the binding attempts in the flow with intensity *λ *are successful. The phrase « *λ *is the parameter of a Poisson stochastic process» is a precise definition of *λ *and its usage in the model.

p. 7. "'Model 1' is referred to when... 'Model 2' is an extension of Model 1..." - if I understand it correctly, 'Model 1' is the module that predicts expression levels given the structure of the locus, set of conditions and all parameters; 'Model 2' is the module that given the desired expression levels at given conditions adjusts the system parameters to maximize the match between the output of 'Model 1' and the input. If so, calling these modules "Models" is probably a misnomer.

#### Authors' response

Corrected. Our computer model can be used in two modes that use different input and output: these are referred to as «Model 1» and «Model 2». An analog can be a motion equation: all masses are given to find trajectories, or all trajectories and some masses are given to find the other masses.

p. 11. "Each transcription factor F is described by a similar stochastic process..." - the role of transcription factors in this model is unclear. How do they interact with polymerases except competing for the binding sites?

#### Authors' response

This interaction is described in the text: a polymerase pushes off any factor and continues to move; if the factor is attached to the binding site the polymerase does not bind and does not affect the factor. Examples of loci with protein factors are known but not provided. Terminators in locus 3 might function as factor binding sites but the authors give reasons in favour of cross-hairpins.

### Reviewer's report 4

Prof. Marek Kimmel

I studied the paper focusing on model description and analysis. In my opinion, the system involving competition of polymerases and other factors for binding sites is quite interesting and the solution may be importance for the applications. Without getting into technicalities, I would like to note two points.

1. Presentation. In the current manuscript, the model can be best understood if the paper is read backwards. It is first stated why the model is needed (which is obviously important), then what are the predictions of the model, and finally how the model is constructed. In my opinion, the clearest way is to describe model's background, then list the hypotheses of the model and the resulting equations (all this as Methods) and finally the results of application of the model, possibly validated using simulated data. Currently, model assumptions are (if I am not missing something) buried within the body of the paper, which makes it difficult to follow.

#### Authors' response

We studied many real genomic loci (only three of them are represented above) and plan to expand into simulations. However, such expansion and reformulation will require further studies. E.g., defining the model assumptions is a separate nontrivial task, now the model per se is an assumption.

2. Mathematical framework. It seems to me that the framework of queuing theory with multiple tasks reaching multiple service stands and competing for service is quite adequate. This framework leads to formulation in the form of Markov chains. Even if the final model involves simplifications (which is quite difficult for me to decide considering the current manuscript), anchoring it in a uniform mathematical formalism seems to make sense.

#### Authors' response

We think that the model suggests a new type of random processes, which is unclear how to study analytically but it would be very interesting even in very special cases; the queuing theory is inapplicable here.
